# Evaluation of How Integrative Oncology Services Are Valued between Hematology/Oncology Patients and Hematologists/Oncologists at a Tertiary Care Center

**DOI:** 10.1155/2018/8081018

**Published:** 2018-04-12

**Authors:** D. M. Hansra, K. McIntyre, J. Ramdial, S. Sacks, C. S. Patrick, J. Cutler, B. McIntyre, K. Feister, M. Miller, A. K. Taylor, F. Farooq, J. Antunez de Mayolo, E. Ahn

**Affiliations:** ^1^Cancer Treatment Centers of America, Atlanta, GA, USA; ^2^Jackson Memorial Hospital, Miami, FL, USA; ^3^Sylvester Comprehensive Cancer Center, Miami, FL, USA; ^4^Miller School of Medicine, University of Miami, Miami, FL, USA; ^5^University of Miami Health System, Miami, FL, USA

## Abstract

Evidence regarding opinions on integrative modalities by patients and physicians is lacking.* Methods*. A survey study was conducted assessing how integrative modalities were valued among hematology/oncology patients and hematologists and oncologists at a major tertiary medical center.* Results.* 1008 patients and 55 physicians were surveyed. With the exception of support groups, patients valued nutrition services, exercise therapy, spiritual/religious counseling, supplement/herbal advice, support groups, music therapy, and other complimentary medicine services significantly more than physicians (*P* ≤ 0.05).* Conclusion*. With the exception of support groups, patients value integrative modalities more than physicians. Perhaps with increasing education, awareness, and acceptance by providers and traditional institutions, integrative modalities could be equally valued between patients and providers. It is possible that increased availability and utilization of integrative oncology modalities at tertiary hospital sites could improve patient satisfaction, quality of life, and other clinical endpoints.

## 1. Background

Evidence shows increased patient utilization of various integrative care modalities in oncology. In part, patients desire greater self-empowerment to manage their symptoms and improve quality of life and overall outcomes. Furthermore with increasing globalization, elements of eastern medicine are widely being recognized and embraced by patients and medical professionals. Measurements of patient and physicians opinions regarding the importance of integrative care are lacking. We aim to compare how National Comprehensive Cancer Network (NCCN) recommended “integrative care” modalities are valued between hematology/oncology physicians and patients.

## 2. Methods

After an institutional review board approval was obtained, a survey study was administered to 1008 hematology and oncology patients and 55 hematologists and oncologists at two outpatient clinical sites at a major tertiary medical center in Miami, Florida, from June 2013 to October 2015. Surveys were administered in the outpatient setting by nursing staff after consent was obtained. Patients filled out surveys in the waiting area; then they were placed in a locked box. Physicians filled out surveys in their office and were placed in a locked box. Both physician and patient surveys were anonymous. The survey was administered in both Spanish and English. The patient survey consisted of 7 questions assessing opinions on integrative care asking: “In addition to standard care, it is important to incorporate/provide the following services in my cancer plan”: (1) nutrition services, (2) exercise therapy, (3) spiritual/religious counseling, (4) supplement/herbal advice, (5) support groups, (6) music therapy, and (7) other complimentary medicine services (acupuncture, massage, and relaxation therapy). Answers were recorded on a 5-point scale (1 = highly disagree, 2 = disagree, 3 = neutral, 4 = agree, and 5 = highly agree) and then converted into 2 categories (1,2, 3 = neutral/disagree versus 4,5 = agree). The physician survey consisted of the same 7 questions assessing opinions on integrative care asking: “In addition to standard care, it is important to incorporate/provide the following services for my patient”: (1) nutrition services, (2) exercise therapy, (3) spiritual/religious counseling, (4) supplement/herbal advice, (5) support groups, (6) music therapy, and (7) other complimentary medicine services (acupuncture, massage, and relaxation therapy). Answers were recorded on a 5-point scale (1 = highly disagree, 2 = disagree, 3 = neutral, 4 = agree, and 5 = highly agree) and then converted into 2 categories (1,2, 3 = neutral/disagree versus 4,5 = agree). Then, Fisher's exact test with two sided *P* value was used to compare the responses between patients and physicians. Demographics collected in the survey included age, gender, race, and ethnicity. Clinical information collected included cancer diagnosis. Inclusion criteria include adult outpatients 18 years old or older with any hematologic or oncologic malignancy. Exclusion criteria include inpatients, patients younger than 18 years old, and those with nonmalignant diagnoses.

## 3. Results

1008 patients were enrolled from June 2013 to October 2015. The mean patient age was 55 years with a range of 18–88 years. 45% of patients were male and 55% of patients were female. 62% of patients were Hispanic versus 38% of patients not Hispanic. 16% of patients were white, 14% black/African American, 2% Asian/Pacific Islander, and 6% other. 21% of patients had hematologic malignancies versus 79% of patients having solid malignancies. 55 physicians were enrolled from June 2013 to October 2015. The mean physician age was 47 years with a range of 29–75 years. 70% of physicians were male and 30% of patients were female. 38% of physicians were Hispanic versus 62% of physicians not Hispanic. 64% of patients were white, 14% black/African American, 11% Asian/Pacific Islander, and 11% other. Survey results were as follows: 84% of patients agree that nutritional advice is important versus 69% of physicians, *P* = 0.0077. 85% of patients agree that exercise therapy is important versus 71% of physicians, *P* = 0.0120. 68% of patients agree that spiritual/religious counseling is important versus 47% of physicians, *P* = 0.0031. 85% of patients agree that advice on supplement/herbal therapies is important versus 44% of physicians, *P* < 0.0001. 60% of patients agree that music therapy is important versus 28% of patients, *P* < 0.0001. 77% of patients agree that “other complementary services” including acupuncture, massage, and relaxation therapy are important versus 47% of physicians, *P* < 0.0001. 71% of patients agree that support groups are important versus 66% of physicians, *P* = 0.4377.

## 4. Discussion

Our study reveals that the majority of patients felt that in addition to standard of care it was important to include nutrition services, exercise therapy, spiritual/religious counseling, supplement/herbal advice, support groups, music therapy, and other complimentary medicine services into their care plan. With the exception of support groups, patients valued integrative modalities more than physicians.

The exact mechanism for the significant discrepancies observed in our study between physicians' and patients' value of integrative modalities is unclear. One major contributing factor may be a physician knowledge gap. Several studies show that physicians currently lack the knowledge, confidence, and training to provide proper guidance to the increasing number of patients who are using integrative modalities [1–6]. A lack of evidence-based information about efficacy, safety, and drug interactions with integrative modalities, as well as a lack of formal training, is thought to be responsible for this deficit [1–6]. In response to increasing use of integrative modalities professional standards are beginning to be issued for pharmacists and physicians and these standards are an important source of guidance with respect to providing patient counseling and utilizing these treatments [2, 5, 7, 8].

The Federation of State Medical Boards (FSMB) has also responded to the increased interest in integrative modalities by issuing its own guidelines [3, 9]. The board recognizes that consistency in standards for evaluating healthcare practices is necessary, whether they are applied to conventional medicine or to integrative modalities [3, 9]. This is highly important as integrative modalities are now increasingly recognized by traditional institutions as having true medicinal value and are held to the same standards as traditional medicine. Furthermore increasing medical schools are offering education in integrative modalities in their curriculums [10]. Perhaps with increasing education, awareness, and acceptance by providers and traditional institutions integrative modalities could be equally valued between patients and providers. Given that providers in traditional institutions, such as tertiary care hospital sites, execute patients' orders, and referrals, it is possible that increased education and awareness of integrative modalities could translate into increased utilization of such services.

In the following sections, we discuss and highlight the evidence behind the integrative modalities with significant disparities.

### 4.1. Exercise

The literature shows that exercise improves quality of life and physical function in patients with cancer [11]. Randomized trials have shown that exercise training is safe, tolerable, and effective for most patients [12–21]. Aerobic and resistance training programs after cancer treatment can improve cardiovascular fitness and strength and can have positive effects on balance, body composition, fatigue, emotional well-being, and quality of life [12–21].

Cancer related fatigue is a common and distressing symptom in cancer patients. Several reviews and meta-analyses of clinical trials show that not only is exercise therapy safe in cancer patients but it also significantly reduces cancer related fatigue [19, 22, 23]. Furthermore, one meta-analysis of clinical trials showed exercise therapy to be superior to pharmaceuticals in reducing cancer related fatigue [24].

Numerous observational studies have consistently found that physical activity is linked to decreased cancer recurrence, incidence, and increased survival for certain tumor types [25–39]. Conversely, inactivity and sedentary behaviors are risk factors for cancer incidence and mortality and also impact mood and quality of life, independent of recreational or occupational physical activity [40–47]. Given the overwhelming evidence all patients should engage in physical activity if possible. Even light physical activity has been shown to improve physical functioning [48]. At minimum patients should be counseled to avoid sedentary behaviors.

### 4.2. Acupuncture

Acupuncture is increasingly used as a therapeutic measure for managing cancer related symptoms. Numerous trials have demonstrated that acupuncture is an effective adjunct in treating cancer related pain [49–52]. Acupuncture has also demonstrated efficacy in relieving side effects of medications such as aromatase inhibitor induced arthralgias [53, 54]. Also, acupuncture may complement other treatments for neuropathic pain by inhibiting various opioid receptors [55]. Acupuncture is also helpful in the treatment and prevention of chemotherapy-induced and postoperative nausea and vomiting [11, 49, 56, 57]. Additionally, acupuncture may also address sleep disturbance, anxiety, drowsiness, and fatigue [56–59] but more research is needed to strengthen the validity of managing other cancer related symptoms [60–62]. Interestingly, acupuncture research has had a twofold higher growth rate compared to biomedical research in the last two decades [63]. Demand for acupuncture treatment in managing cancer outcomes has increased [64] in tandem with increased research.

### 4.3. Music Therapy

There are many benefits of music therapy for patients including improving mood, decreasing stress, pain, anxiety level, and enhancing relaxation [65]. When used in conjunction with conventional cancer treatments, music therapy has been found to help patients promote a better quality of life [66]. Music therapy includes both passive and active forms. Passive music therapy entails a patient listening to live or recorded music whereas active music therapy techniques include engaging the patient in singing, music composition, and instrument playing.

One study examined 51 studies on 1867 patients exposed to passive music therapy and evaluated its effects in both children and adults [67]. The results showed that listening to music reduced not only pain intensity levels but also opioid requirements [67]. One large systematic review of over 200 randomized controlled trials (RCT) showed that passive music therapy improves depression and anxiety in female patients with breast cancer [68].

The data on active music therapy in cancer patients is less robust than passive music therapy. One prospective study in terminally ill cancer patients utilizing components of active music therapy showed that the majority of patients experienced a positive benefit [69]. In addition to music therapy other forms of art therapy have been shown to be of benefit in cancer patients. For example, one systematic review and meta-analysis of clinical trials that included clinical trials utilizing dance and movement therapy and art therapy working with visual arts materials have beneficial effects on anxiety in patients with breast cancer [70].

Music can be easily delivered to patients in a variety of formats such as live or recorded concerts and performances on tapes, video, and mobile devices. Also music therapy can easily be delivered in any setting such as the comfort of one's own home, chemotherapy infusion suite, or hospital room. Furthermore, other forms of art therapy such as active music therapy and visual art therapy are safe, relatively inexpensive, and with no risk of systemic side effects to the patient. Due to the favorable risk/benefit profile all patients should be offered music therapy and art therapy as a part of their cancer care.

### 4.4. Herbal Medicine, Vitamins, and Mineral Supplements

Evidence shows increased utilization of herbal medications among cancer patients [71, 72]. There are numerous herbal medications that claim to have anticancer effects but in general high quality clinical evidence is lacking. There is good evidence showing antitumor activity in laboratory studies for a variety of herbal compounds. For example, green tea* (Camellia sinensis)* and Mistletoe* (Viscum album)* have demonstrated antineoplastic effects such as antiproliferative or apoptosis-inducing in various cancer cell lines [73–81]. Also Graviola* (Annona muricata)* has been shown to inhibit tumorgenicity by inhibiting multiple signaling pathways in pancreatic and breast cancer cell lines [82].

Herbal medications have also been used to manage side effects. For example, Mistletoe has been well studied and several reviews and clinical trials have shown that the application of Mistletoe extract has been associated with a reduction of chemotherapy-related side effects as well as an improvement in quality of life during chemotherapy [81, 83–88]. Ginger has been used to relieve chemotherapy-induced nausea and vomiting [89–91]. Also, St. John's Wort* (Hypericum perforatum)* is utilized for antidepressant properties [92], and Kava kava* (Piper methysticum)* was found to decrease anxiety in meta-analyses of multiple randomized trials [93, 94]. Though patients often perceive herbal medicine as more natural and hence safer than other more conventional treatments, concerns about their safety and potential risks remain [72, 95]. Kava kava was withdrawn after reports of hepatotoxicity and liver failure secondary to its use emerged [96]. In addition, inadequate quality control of these products may give rise to contamination of the herbs with heavy metals, pesticides, and so forth [72]. For example,* PC-SPES*, a mixture of herbs, was found to have considerable antineoplastic effects [71, 97–99], but it was later withdrawn when reports emerged that it may be contaminated with Warfarin and may induce bleeding diathesis [99, 100].

Drug-herb interactions also remain of great concern regarding potential harmful interactions and increased toxicity, as well as decreased efficacy of prescribed treatments [72, 95]. For example,* Ginkgo biloba* and* Ginseng (Panax ginseng)* are CYP3A4 and CYP2C19 inhibitors and thus might interact with a variety of chemotherapy agents.

It is estimated that 79–85% of cancer survivors take vitamin or mineral supplementation, often without disclosing this information to their providers [101–103]. Of concern, an analysis of dietary supplements found that many supplements contain unlisted ingredients or banned pharmaceutical ingredients [32, 104] which could be potentially toxic to patients. No clear evidence supports an effect of dietary supplements for cancer prevention, control, or recurrence [32, 105, 106]. Given the high percentage of patient's usage of herbal medication and supplements, we recommend that providers should assess all herbal medication and supplement use and prescribe supplements in patients only with documented or at risks deficiencies. If patients desire usage of herbal medications, they should be referred to an integrative certified specialist or oncologist with experience in this area. Furthermore patients with interest in taking herbal medications and supplements should be encouraged to participate in clinical trials when indicated.

### 4.5. Massage Therapy

Evidence shows massage therapy benefits cancer patients by relieving symptoms and improving well-being [107]. A study of 1290 patients treated with massage therapy experienced a 50% reduction in symptom scores for fatigue, nausea, and anxiety [108]. The same study additionally found that the effects of massage therapy were greater for outpatients, with a 10% increase in improvement over inpatients [109]. Separate RCTs have also demonstrated that massage therapy leads to reductions in pain, nausea, fatigue, perceived stress, and cortisol levels in breast cancer patients undergoing chemotherapy [109–111]. Furthermore, massage therapy has been shown to decrease depression, reduce mood disturbances, and anger in patients [110, 112, 113]. Also, one meta-analysis of 18 RCTs including 950 patients showed significant favorable effects on fatigue and negative emotions [114]. Reviews studying the outcomes of massage therapy have found that it may reduce pain and anxiety short-term; however the long-term benefits remain unclear [107, 115, 116]. With regard to implementation of massage therapy, a recent study at chemoinfusion centers found that massage can be safely administered at chemoinfusion suites [117]. Given the excellent benefit-risk ratio of message therapy, this modality should be offered as indicated to interested patients.

### 4.6. Nutrition

Numerous studies have shown that various aspects of nutrition are important in cancer patients. Some studies focus on the importance of avoiding malnutrition and assessing nutritional status in cancer patients [118]. For example, it has been shown that early identification of nutritional deficiencies can improve quality of life and survival in patients with non-small cell lung cancer (NSCLC) and that malnutrition is an independent prognostic factor in advanced NSCLC [119].

Nutrition has also been shown to play an important role in the perioperative setting. For example, assessments of nutritional status and use of prognostic indices can help predict postoperative complications and clinical outcomes in patients with gastrointestinal cancers [119]. Additionally, the preoperative prognostic nutritional index (PNI) is an independent predictor of survival in patients with renal cell carcinoma and gastric carcinoma [120, 121].

Larger studies have focused on outcomes related to nutritional intake in cancer patients. Studies have shown that healthy dietary patterns are associated with decreased risk of cancer development overall [121, 122]. A study in England with >65000 participants showed that at least 7 servings per day of fruits and vegetables decreased cancer incidence by 25% [123].

Studies have also shown that healthy dietary patterns are associated with a decrease in cancer recurrence and improved outcomes in survivors. For example, in survivors of stage III colon cancer, a diet with more fruits, vegetables, chicken, and fish showed improved outcomes in terms of cancer recurrence and overall survival [124]. High dietary glycemic load has also been associated with increased risk of recurrence and mortality in survivors, as was high intake of red and processed meat [125, 126]. Also, meta-analyses have shown that whole grain intake is inversely associated with cancer mortality [127].

Evidence also exists for tailored nutrition to specific cancers. For example, omega 3 polyunsaturated fatty acids (PUFAs) have been shown to be beneficial after surgery performed for gastric cancer, have been shown to play a role in inflammation in Colorectal Cancer [128], and have been shown to improve outcomes and prognosis in pancreatic cancer patients [129]. Additionally, a study of 4282 women suggested that an olive oil/Mediterranean diet reduced the risk of invasive breast cancer [130].

Of importance, the interrelation between obesity and nutrition plays an important role in cancer outcomes. It has been shown that weight gain or being overweight or obese can worsen a survivor's risk for cancer recurrence or death. For example, a meta-analysis showed that there is a correlation between BMI and higher risk of total and breast cancer specific mortality [131]. Another study showed that survivors of stage II and III colon cancer enrolled in NSABP trials from 1989–1994 with a BMI of 35 or greater had an increased risk of disease recurrence and death [132]. Furthermore, there have been studies showing that weight loss or gain increases mortality risk in survivors suggesting that weight maintenance is optimal [133].

Per current evidence and NCCN guidelines, the following is recommended: in terms of food volume, fruits and vegetables should take up half of the volume on the plate (30% vegetables, 20% fruit), whole grains should be 30% of the plate, and protein should be 20% of the plate. In terms of food components, fat in the diet should be obtained from plant oils, avocados, seeds, nuts, and fatty fish. Carbohydrates should be obtained from fruits and vegetables, legumes, and whole grains. Proteins should be obtained from poultry, fish, legumes, low fat dairy foods, and nuts. In addition, limited intake of red or processed meats and refined sugars is encouraged. Referral to a Certified Specialist in Oncology Nutrition (CSO) if available is ideal [134]. Furthermore, overweight/obese patients should discuss nutrition with their providers, specifically portion control and tracking calories to normalize BMI. Those who are underweight should increase frequency of eating and avoid fluid intake with meals [135].

### 4.7. Spiritual and Religious Support

Spiritual and religious support has become increasingly important component of supportive care for cancer patients as this is associated with improved mental health in cancer patients [136] and quality of life [137–139]. Also, attendance at religious services is associated with lower cancer related mortality [140]. Ideally, religion and spirituality can aid patients in identifying positive coping skills, managing grief [141], finding new goals, redefining hope, and realizing purpose and peace in suffering [142]. In our study, a majority of patients (68%) felt that religious and spiritual counseling was important to their cancer care which is consistent with one study by Balboni et al. showing a majority of patients (88%) considered religion important to their care [143]. In the same study, 72% of patients reported receiving little to no support from their medical system [143]. Religious and spiritual care results not only in clinical improvements but also in customer satisfaction [144]. Although there are mixed reports on outcomes from religion and spirituality studies, there is overwhelming evidence that demonstrates positive clinical effects [145–150]. All patients should be assessed for religious and spiritual related distress and be referred to a chaplaincy professional upon request.

## 5. Study Limitations

Our study has several limitations. First, survey studies are prone to response bias. In terms of demographics, our patient population was predominantly Hispanic and white; therefore caution should be used in extrapolating our data to the general hematology/oncology populations. Last, our study was conducted in South Florida which has unique sociocultural influences compared to other states so again extrapolation of this study to the general cancer population is cautioned.

## 6. Study Strengths

Our study enjoyed several strengths including a large number of patients (over 1000) and a large number of physicians (over 50) and the power of the study was adequate for confident analysis. Furthermore, we followed strict guidelines to maintain anonymity among participants to minimize bias. Also, this is the first and largest study addressing opinions integrative modalities in both hematology oncology patients and providers. Although the patient population was predominately white Hispanic, there was a significant amount of population heterogeneity included in this study. Last, the physician population was heterogeneous which likely reflects general hematologist oncologist population in the United States.

## 7. Conclusions

The majority of patients feel that in addition to standard of care it is important to include nutrition services, exercise therapy, spiritual/religious counseling, supplement/herbal advice, support groups, music therapy, and other complimentary medicine services such as acupuncture, massage, and relaxation therapy into their care plan. With the exception of support groups, patients value integrative modalities more than their physicians. Since physicians formulate and execute patient care plans, it is important that they recognize these modalities as desired by the majority of patients and also that these modalities are endorsed by the NCCN as category 1 and 2A recommendations. Addressing these major disparities between physician and patients should be a top priority for tertiary care sites. Perhaps with increasing education, awareness, and acceptance by providers and traditional institutions, integrative modalities could be equally valued between patients and providers. Furthermore, it is possible that increased availability and utilization of integrative oncology modalities at tertiary hospital sites could improve patient satisfaction, quality of life, and other clinical endpoints.
